# IDH1/IDH2 Inhibition in Acute Myeloid Leukemia

**DOI:** 10.3389/fonc.2021.639387

**Published:** 2021-03-29

**Authors:** Claudio Cerchione, Alessandra Romano, Naval Daver, Courtney DiNardo, Elias Joseph Jabbour, Marina Konopleva, Farhad Ravandi-Kashani, Tapan Kadia, Maria Paola Martelli, Alessandro Isidori, Giovanni Martinelli, Hagop Kantarjian

**Affiliations:** ^1^Hematology Unit, Istituto Scientifico Romagnolo per lo Studio e la Cura dei Tumori (IRST) IRCCS, Meldola, Italy; ^2^Dipartimento di Chirurgia e Specialità Medico-Chirurgiche, Sezione di Ematologia, Università degli Studi di Catania, Catania, Italy; ^3^Hematology and Clinical Immunology, University of Perugia, Perugia, Italy; ^4^Hematology and Stem Cell Transplant Center, AORMN Hospital, Pesaro, Italy; ^5^Leukemia Department, MD Anderson Cancer Center, Houston, TX, United States

**Keywords:** acute myeloid leukemia, AML, enasidenib, IDH, isocitrate dehydrogenase, ivosidenib, target therapy

## Abstract

Recently, the discovery of biological and clinical properties of mutated isoforms 1 and 2 mutations of isocitrate dehydrogenases (IDH) 1 and 2, affecting approximately 20% of patients with acute myeloid leukemia (AML), lead to the development of an individualized treatment strategy. Promoting differentiation and maturation of the malignant clone targeting IDH is an emerging strategy to promote clinical responses in AML. Phase I/II trials have shown evidence of safety, tolerability, and encouraging evidence of efficacy of two small molecule inhibitors targeting IDH2 and IDH1 gene mutations, respectively enasidenib and ivosidenib. In this review, the contribution of IDH1/IDH2 mutations in leukemogenesis and progress of targeted therapeutics in AML will be highlighted.

## Introduction

In the last ten years, advances in deciphering genomic landscape have increasingly contributed to the refinement of prognostication in acute myeloid leukemia (AML), incorporating the mutational status of some genes (1–4) in the WHO classification of AML.

The current (2017) European Leukemia Net (ELN) classification recommends the stratification of newly-diagnosed AML based on the mutational status of five genes *NPM1*, *CEBPA*, *FLT3*, *ASXL1*, *TP53*, and *RUNX1* (2). Indeed, mutation in both *NPM1* and *CEBPA* genes have been associated with a favorable prognosis, in the absence of a concomitant internal tandem duplication within the *FLT3* gene (*FLT3*-ITD) in *NPM1* mutated cases. Moreover, mutational load appears to be an additional critical factor, which is why the ELN recommends a cut-off of 0.5 for the allelic ratio of mutant to wildtype *FLT3*. *RUNX1*, *TP53*, and *ASXL1* mutations all confer an unfavorable prognosis as do *FLT3*-ITD mutations (at a high allelic ratio of > 0.5 in the absence of *NPM1* mutations) (2).

Despite the improvements in understanding its genomic basis, AML is still a therapeutic challenge for older adults, not eligible to allogeneic stem cell transplantation, with less than 10% chance of long-term survival (2, 5). The therapeutic scenario of AML is transitioning from a standard chemotherapy regimen in all patients toward individualized therapeutic strategies (6–9).

The discovery that some AML blasts and glioma cells carry specific mutations in isocitrate dehydrogenase isoform 1 (IDH1) and 2 (IDH2), resulting in the generation of a particular metabolite — the (R)-enantiomer of 2-hydroxyglutarate ((R)-2HG), led to development of small molecular inhibitors which effectively inhibit 2HG production; effectively targeting the aberrant metabolism of AML blasts (10).

Enasidenib and ivosidenib, two small molecule inhibitors targeting *IDH2* and *IDH1* gene mutations, respectively, have been recently approved by FDA for their efficacy and safety. This review highlights recent advances on metabolic deregulation in AML and novel strategies for tailored therapy targeting mutations in IDH1 and IDH2.

## Metabolic Rewiring and Epigenetic Aberrancies in AML Blasts Carrying IDH1/IDH2 Mutations

AML arises from genetic abnormalities in hematopoietic stem or progenitor cells, responsible of uncontrolled growth and accumulation of neoplastic blasts in the bone marrow leading to organ failure and often death. Somatic mutation in *IDH1* and *IDH2* occur respectively in about 6%–16% and 8%–19% of AML patients (11), are frequently associated with normal karyotype and *nucleophosmin* (*NPM1*) gene mutations (12), and are without a clear prognostic relevance (13), unless their increased frequency with advanced age (2, 3, 14–16).

In AML patients, *IDH1*/*IDH2* gene mutations are heterozygous missense mutations involving a single arginine (R) residue in the enzyme active site (9), R132 in *IDH1* and R140 or R172 in *IDH2*, restructuring the enzyme and leading to a reduced affinity of the mutant enzymes for isocitrate while increased affinity for α-ketoglutarate (αKG) and nicotinamide adenine dinucleotide phosphate (NADPH) with production of R-2HG (17), as extensively reviewed elsewhere (18).

7Under physiological conditions the IDH3 isoform generates NADH as the canonical product of the Krebs cycle, while IDH1 (the isoform localized in the cytoplasm and peroxisomes) and IDH2 (the isoform localized in mitochondria) catalyse the oxidative decarboxylation of isocitrate to generate αKG and carbon dioxide (CO_2_) and to produce reduced NADPH from NADP+, playing a critical role for the maintenance of the intracellular reduced glutathione pool and preserving cellular homeostasis (19). Mutants of IDH1/2 redirect carbon metabolites away from the Krebs cycle and oxidative catalyzing the conversion of α-ketoglutarate (α-KG) to D-2-hydroxyglutarate (D2HG), promoting glutaminolysis pathway, to provide carbon skeletons required for anaplerosis to fuel the Krebs cycle (20) and reduce the enzymatic activity of the wild-type isoforms (20).

mIDH1/2 cells still need αKG to produce D-2HG, even if on the other hand they restrict αKG synthesis as consequence of reduced glycolytic influx and impairment of Krebs cycle (20).

Moreover, 2DHG acts as an oncometabolite through a variety of mechanisms.

First, D2HG directly inhibits α-KG-dependent enzymes such as the ten-eleven translocation (TET) family enzymes (21).

Second, D2HG accumulation additionally causes genetic instability and contributes to tumorigenesis, either directly by inhibiting α-KG-dependent alkB homolog (ALKBH) DNA repair enzymes or indirectly by altering the expression of DNA repair genes (22). Uncontrolled increase of D2HG levels inhibits αKG-dependent lysine demethylases in a competitive manner, thus to increase histone methylation in a variety of cell line models (22–24). Further D2HG-mediated inhibition of cellular differentiation promotes in turn the pathological self-renewal of stem-like progenitor cells, favoring the malignant transformation (19, 25). Additional changes in the epigenetic machinery of hematopoietic progenitors can occur *via* several other molecular mechanisms, that could explain why IDH1 and IDH2 mutations are mutually exclusive with Tet methylcytosine dioxygenase 2 (TET2) mutations (26).

*IDH1* mutations are found in almost all FAB (French-American-British classification) AML subtypes, associated to elderly age, diploid or intermediate cytogenetics, increased platelet counts in mutated cases (27). Experimental observations collected *in vitro*, where *IDH2* mutants can block the differentiation of HSPCs (26, 28, 29) and *in vivo* suggest that *IDH1/IDH2* mutations act as canonical class II mutations, required to cooperate with class I mutations to promote AML.

Conditional knock-in mice carrying *IDH1^R132H^* mutation have a characteristic *hypermethylation signature*, similar to that one observed in human IDH1- or IDH2-mutant AML. Mutant mice do not develop leukemia and had a similar survival as wild-type mice, but they display a myeloproliferative phenotype, characterized by increased number of early hematopoietic progenitors, splenomegaly and anemia with extramedullary hematopoiesis, along with a partial blockage in myeloid differentiation (28).

In hematopoietic stem and progenitor cells (HSPCs) obtained from two mouse models incorporating two common class I mutations observed in human AML, FLT3-ITD and Nras^G12D^, IDH2^R140Q^ or IDH2^R172K^ mutations conferred inferior overall survival and drive aggressive AML. mIDH2^R172^ murine AMLs displayed the histopathological and molecular features of the human disease, including chemoresistance phenotype associated to poor patient survival (29).

In mice, *mIDH2^R140Q^* in hematopoietic tissues was not sufficient for the development pf AML phenoype, suggesting the requirement of additional driver mutations, like overexpression of HoxA9 and Meis1a or mutations in FMS-like tyrosine kinase 3 (FLT3) to drive acute leukemia *in vivo* (30).

Combining the loss-of-function due to miRNA Mir142 with *IDH2^R140Q^* mutation *in vivo*, only recipients of double mutant cells developed fatal leukemia, since HOXA cluster genes in myeloid progenitors were alleviated by Mir142 loss-of-function (31).

Thus, IDH1/IDH2 mutations play a double role in leukemogenesis: first, mutants induce alterations in the pattern of histone modifications and aberrant DNA methylation, with consequent accumulation of epigenetic aberrancies, associated with reduced TET2 activity (26); second, mutants can rewire metabolism of AML blasts (25), with sustained inhibition of the activity of cytochrome c oxidase (COX) in the mitochondrial electron transport chain (20) and the glutamine addiction of AML cells for survival (32). Small molecule inhibitors of mIDH1 and mIDH2, and vitamin C supplementation [cofactor of TET2 proteins (33, 34)] can abrogate the production of 2HG or enhance TET2 activity, respectively, restoring DNA methylation patterns and myeloid cell differentiation (35).

The same pathways can be elicited by metabolic derangements in IDH1/IDH2 wild-type blasts. For example, overexpression of branched chain amino acid in leukemia cells decreased intracellular αKG levels and caused DNA hypermethylation through altered TET activity, leading to a DNA hypermethylation phenotype similar to cases carrying an IDH mutant (36).

## Targeting IDH1 Mutants in AML: Ivosidenib

Ivosidenib (Tibsovo; Agios Pharmaceuticals, Inc.; formerly AG-120) is the first-in-class, selective, allosteric *IDH1^R132^* inhibitor (37), derived from AGI-5198, which could not enter clinical studies due to its poor pharmaceutical properties (37). *Ex vivo*, AG-120 can induce differentiation of primary *mIDH1* blasts obtained from AML patients, as shown by enhanced ability to form differentiated colonies in methylcellulose assays, increased levels of cell-surface markers of differentiation, and increases in the proportion of mature myeloid cells (37). *In vivo*, in a human IDH1-mutated tumor xenograft model it showed potent activity in lowering tumor levels of R-2-HG, good pharmacokinetics properties and was well tolerated (37).

### Clinical Efficacy

Ivosidenib was approved by the U.S. Food and Drug Administration (FDA) for patients with relapsed or refractory IDH1-mutated AML in 2018 (38), and also as a front-line therapy for newly diagnosed elderly patients 75 years or older or who are ineligible to receive intensive chemotherapy in 2019, based on promising results of phase I-II clinical trials (39).

The single-arm trial AG120-C-001 first established the efficacy of ivosidenib on the basis of complete remission (CR) + CR with partial hematologic recovery (CRh) rate, duration of CR + CRh, and conversion from transfusion dependence (TD) to transfusion independence (TI). With median follow-up of 8.3 months for 174 adults with IDH1-mutated relapsed/refractory (R/R) AML treated with 500 mg ivosidenib daily, the CR + CRh rate was 33% [95% confidence interval (CI), 26-40], median duration of response was 8.2 (95% CI, 5.6-12) months, and conversion from TD to TI occurred in 37% of patients (38).

In the phase I dose escalation and dose-expansion study including 258 patients with *IDH1*-mutated hematologic malignancies, ivosidenib was administered orally, daily, in 28-day cycles. The 500mg dose was selected for the dose-expansion phase since higher doses did not demonstrate enhanced inhibition and further R-2-HG suppression (40). Among the 125 R/R AML patients in the primary efficacy population, overall response rate (ORR), continuous complete remission (cCR), and complete remission (CR) rates were 41%, 30%, and 22%, respectively. Median time to cCR was 2.7 months and median cCR duration was 8.2 months. After a median follow-up of 14.8 months, the median OS was 8.8 months and for cCR patients the 18-month survival was 50%. Molecular remission, defined as mutation clearance, was observed in 21% of patients with CR and was associated with longer OS (40).

In the setting of newly diagnosed *IDH1*-mutated AML (76% with secondary AML) patients ineligible for intensive induction therapy, ivosidenib given at dose of 500 mg/daily as single agent demonstrated cCR+CR and CR rates of 42.4% and 30%, respectively, associated with transfusion independence in 42.9%. Median durations of cCR and CR were not reached with a median follow-up of 23.5 months. The median OS for all patients was 12.6 months. Notably, *IDH1* molecular remission, analyzed by NGS on BM and PB samples, was observed in 4/4 and 5/10 patients achieving a cCR and CR, respectively (41).

### Predictors of Response to Ivosidenib

No single gene-mutations have been significantly associated with clinical response to ivosidenib. The observation that baseline *IDH1* mutated variant allele frequency (VAF) and 2DHG did not significantly impact the achievement of a complete response prompted researchers to investigate additional predictors of response. In the phase 1, multicenter, open-label, dose-escalation, and dose-expansion study of ivosidenib NCT02074839, involving 179 patients, mutations in receptor tyrosine kinase (RTK) pathways (including NRAS, KRAS, PTPN11, KIT, and FLT3) were associated with a lower likelihood of clinical response to ivosidenib monotherapy in R/R AML. There was no association between clonal or subclonal mIDH1 status (defined as co-mutation variant allelic frequence, VAF, greater 5%) and achieving a best response of CR.

Acquired resistance was mediated *via* diverse mechanisms, including emergence or expansion of AML-related mutations in RTK and 2-HG–restoring pathways (comprising second-site mutations in *IDH1* and mutations in *IDH2*), not mutually exclusive within an individual patient, resulting in increased 2-HG (42). However, co-occurring mutations in *ASXL1*, *RUNX1*, *TP53* and *JAK2* genes, commonly associated with a worse prognosis in AML, did not significantly affect the clinical response rate to single agent ivosidenib (42).

### Adverse Events and Safety in Patients Treated With Ivosidenib

Overall, both in R/R and newly diagnosed AML patients, oral ivosidenib was well tolerated and associated mostly with manageable AEs. Grade ≥ 3 AEs were experienced by 21% and 79% of R/R AML and ND AML patients, respectively. The most relevant adverse events were QTc prolongation and differentiation syndrome (DS), associated to with rapid proliferation and differentiation of myeloid cells that may be life-threatening or fatal if not treated. QT prolongation was observed in 25% R/R AML and 18% newly diagnosed AML patients, respectively, but no patients needed permanently discontinuation of ivosidenib (40, 41). Ivosidenib should be interrupted if QTc increases to greater than 500 ms and permanently discontinued in patients who develop QTc interval prolongation with signs or symptoms of life-threatening arrhythmia.

Other adverse events included gastrointestinal (diarrhea, nausea, abdominal pain, constipation), Guillain-Barré syndrome and hematological (anemia, thrombocytopenia, leukocytosis) mild adverse events (40, 41).

Although the ivosidenib/azacitidine combination could be safely administered in an outpatient setting, more than half of the patients required hospitalizations related to AEs. However, the number of hospitalization days per patient-year of drug exposure owing to AEs in this study was encouragingly lower than that previously reported for azacitidine monotherapy (43).

### Differentiation Syndrome Management in Patients Treated With Ivosidenib

In the clinical trial setting, 25% (7/28) of patients with newly diagnosed AML and 19% (34/179) of patients with R/R AML treated with ivosidenib experienced DS, from as early as 1 day to up to 3 months after treatment initiation (44). Median time of onset was 29 days (range 5 – 59) and 14.5 days (8 – 82) in R/R and ND patients in clinical trials (40, 41, 44), while in other series median time to onset of DS has been reported as 20 days (range 1 – 78) (45). DS symptoms included noninfectious leukocytosis, neutrophilia and constitutional manifestations, including fever, peripheral edema, pyrexia, dyspnea, pleural effusion, hypotension, hypoxia, pulmonary edema, pneumonitis, pericardial effusion, rash, fluid overload, tumor lysis syndrome, and increased creatinine. Of the 34 R/R AML patients who experienced DS, 27 (79%) patients recovered after treatment of DS or after dose interruption of ivosidenib (40, 41).

In a recent systematic analysis of DS in R/R AML patients treated with ivosidenib and enasidenib on behalf of the US FDA, a higher relative risk of DS was associated with peripheral blast count ≥25%, bone marrow blast count ≥48%, and concurrent *TET2* mutations (45).

DS therapy consisted of corticosteroids (e.g. dexamethasone 10 mg IV every 12 h, or an equivalent dose of an alternative oral or IV corticosteroid) for a minimum of 3 days, diuretics and cytoreduction if leukocytosis was associated. Symptoms of DS may recur with premature discontinuation of corticosteroid and/or hydroxyurea treatment. Repeated DS occurred in 12% of patients. If severe signs and/or symptoms persist for more than 48 h after initiation of corticosteroids, ivosidenib interruption is recommended until signs and symptoms of DS are no longer severe (44).

## Targeting IDH2 Mutants in AML: Enasidenib

Enasidenib (Idhifa; Celgene Corp.; formerly AG-221) is the first-in-class, orally available, small molecule, selective inhibitor of mIDH2. AG-221, developed by Agios Pharmaceuticals in partnership with Celgene, is specific for mutants *IDH2^R140Q^* and *IDH2^R172K^*, by binding to its allosteric site and stabilizing the homodimer conformation preventing the conformation change required for its catalytic action and the production of the R2HG (46).

Preclinical studies conducted *in vitro* and *in vivo* showed that AG-221 could induce differentiation due to increased granulation, phagocytic activity and expression of mature myeloid markers with concomitant changes in global DNA hypermethylation, although retention of mIDH2 in differentiated cells. Survival benefit in primary human *IDH2*-mutated AML xenograft mice compared to mice treated with cytarabine was dose-dependent, associated with reduction in R-2-HG, providing strong rationale for a phase I/II clinical trials (46).

### Clinical Efficacy

The phase I/II, multicenter, multinational, dose-escalation and expansion clinical trial enrolled 239 patients with advance myeloid malignancies, mostly R/R AML, to establish pharmacokinetic and pharmacodynamic profiles of enasidenib along with evaluation of clinical efficacy (47).

In the initial dose escalation part of the study, 113 patients were administered doses of enasidenib ranging from 50 to 650 mg continuously daily in 28-days cycles and no MTD was identified. The dose chosen for the expansion phase was 100 mg daily based on its pharmacokinetic and pharmacodynamic analyses (maximized plasma depletion of R-2-HG). Thus, 119 R/R AML patients (including 32% refractory to initial induction, 23% who had relapsed within 1 year of treatment, and 11% who relapsed after prior stem cell transplant), median age of 67 years old (range: 19–100), received enasidenib at 100 mg daily in 28-days cycles. Most patients (76%) carried the *IDH2^R140^* mutation (47).

The overall response rate, for all R/R AML patients was 40.3%, including 34 patients (19.3%) who achieved CR. Median time to first response was 1.9 months. Enasidenib was active against both types of *IDH2* mutations, with patients with *IDH2^R172^* showing an improved ORR compared with patients with *IDH2^R140^* mutations, 53.3% vs 35.4%, respectively, although CR rate was not significantly different. The median duration of response was 5.8 months and median overall survival (OS) in patients with R/R AML was 9.3 months (8.2–10.9 months) with an estimated one-year survival of 39%. In patients who achieved a CR, the median OS was 19.7 months (47, 48).

### Predictors of Response to Enasidenib

As shown in previous preclinical studies, the amount of the oncometabolite R-2-HG was decreased upon enasidenib treatment with a median suppression of 90.6%, although its reduction was not clearly associated with clinical outcome and could not be considered a reliable biomarker. Reduction of R-2-HG was higher in patients carrying *IDH2^R140^* than *IDH2^R172^* mutant and was not associated with likelihood of response (48) (49). Additionally, *IDH2* mutational burden measured by variant allele frequency (VAF) did not correlate with clinical outcome and therefore should not be used for treatment decisions with enasidenib.

Enasidenib induced responses in ∼40% of patients with mutant-IDH2 R/R AML, with similar response rates regardless of response to prior treatment. However, preliminary data suggest that molecular remission, defined as defined as IDH2 VAF below the limit of detection (0.02% to 0.04%) at ≥1 time point, was associated with attainment of complete remission with enasidenib (48).

*IDH2* mutant allele persist in patients despite long treatment and clinical response (48, 49), supporting that enasidenib *in vivo* promotes differentiation of leukemic blasts, providing the rationale to develop combinations with chemotherapy to promote blasts clearance (50).

Patients who experienced differentiation syndrome (DS) had higher CR (36.4%) than those who did not have DS (CR 26.2%) (51). However, in independent analysis, response rates, duration of response, and overall survival were lower in patients with versus without DS, probably due to less frequent dose intensity (45).

Failure to respond to enasidenib was associated with NRAS mutations, and lower mIDH2 VAF among IDH2-R172 patients. In patients carrying more than six mutations co-occurring with *IDH2* were less likely to achieve a response with enasidenib (48). In a substudy of 37 paired samples from a cohort of 176 patients enrolled in a clinical trial, several mechanisms underlying acquired resistance to IDH2 inhibition have been identified, which seldom occur by second site mutations in *IDH2*, but by multiple mechanisms through clonal evolution or clonal selection involving:

1) *IDH1* acquired mutations (R132C, R132H);
2) cytokine receptor signaling mutations (*CSF3R*, *FLT3*);
3) hematopoietic transcription factors mutations (*RUNX1*, *BCL11A*, *GATA2*);
4) spliceosome factors mutations (*DDX1*, *DHX15*);
5) chromosome 7 deletions;
6) mutations in genes recurrently mutated in hematopoietic cancer (*ELMO3, NFKB1, BCOR, CACN1G, UGT2B10, BRCA2, SCN3A, SETD1B, AKAPBL, PLCL1, DEAF1*) and 7) in other cancer (*SLC1BA3, IL17A, MTUS, DOM3Z*).

Surprisingly, in most patients, R-2-HG levels remained suppressed at relapse, indicating that disease recurrence was not relying on IDH2 mutant activity; moreover, RTK pathway genes mutations were not associated with acquired resistance in this group of patients with *IDH2*-mutated R/R AML (52).

In a few cases, therapeutic resistance can be associated with the emergence of second-site IDH2 mutations in trans, interfering with its allosteric enzyme inhibition and preserving R-2-HG synthesis, as shown in an ivosidenib resistant patient (53). The expression of either of these mutant disease alleles alone did not induce the production of 2HG *in vitro*; however, the expression of the Q316E or I319M mutation together with the R140Q mutation in trans allowed 2HG production that was resistant to inhibition by enasidenib (53).

The ongoing IDHENTIFY (ClinicalTrials.gov, NCT02577406) international, multicenter, phase 3 randomized, open-label trial is ongoing to compare the efficacy of enasidenib to conventional care (including best supportive care only, azacitidine subcutaneously plus best supportive care, low-dose cytarabine subcutaneously plus best supportive care, or intermediate-dose cytarabine intravenously plus best supportive care) in 319 patients, older than 60years, with advanced R/R *IDH2*-mutated AML. The study missed the primary endpoint (overall survival in experimental arm) and has been declared negative, but publication of final results is still expected.

In first line, the phase II portion of an open-label, randomized phase I/II study (NCT02677922) comparing 75 mg/m2/day × 7 day/cycle azacytidine alone or in combination with enasidenib 100 mg QD is currently under investigation. Preliminary data showed that the enasidenib plus azacytidine combination resulted in significantly improved response rates and durations, and was generally well-tolerated in older patients with mIDH2 newly diagnosed AML (54). In particular, the ORR was 48% with combination therapy compared to 14% with azacitidine alone, with a median OS was 22 months in both of the treatment arms and longer EFS in the combination therapy arm (54).

Ongoing trials are investigating the use of enasidenib single agent as maintenance therapy after salvage induction chemotherapy (ClinicalTrials.gov, NCT03881735) or post allogeneic hematopoietic stem cell transplantation in patients harboring the *IDH2* mutation (ClinicalTrials.gov, NCT03515512 and NCT03728335).

### Adverse Events and Safety in Patients Treated With Enasidenib

Among all 345 patients enrolled in the phase I/II study, the most common grade 3 or 4 treatment-related adverse events were indirect hyperbilirubinemia (10%), thrombocytopenia (7%), and IDH differentiation syndrome (6%) (48).

In the setting of 39 elderly newly-diagnosed patients, the three most common adverse events experienced were anemia, increased levels of indirect hyperbilirubina and differentiation syndrome. Unfortunately, 21% of the patients experienced serious adverse events including tumor lysis syndrome (55).

### Differentiation Syndrome Management in Patients Treated With Enasidenib

Based on Montesinos criteria (56), AG-221 investigators identified 33/281 (12%) cases of DS in *IDH2*-mutated R/R AML patients treated with enasidenib in the first pivotal phase 1/2 study (ClinicalTrials.gov, NCT01915498). Most frequent symptoms included dyspnea, fever, hypoxia, and pulmonary infiltrates, beginning with a median time of 30 days (range 7-129).

As described above for the management of DS in patients treated with ivosidenib, corticosteroid therapy, hydroxyurea and temporary enasidenib discontinuation were effective in improving DS symptoms; no deaths were reported (51).

In an independent analysis, median time to onset of DS was 19 days (range 1 – 86). A higher relative risk of DS was associated with peripheral blast count ≥15% and bone marrow blast count ≥48% for both groups. Higher RR of DS was also observed with a higher median WBC count, serum LDH and prior HSCT and concurrent mutations in *SRSF2*. Repeated DS occurred in 15% of patients (45).

Although differentiation phenomenon upon IDH1 or IDH2 inhibitors is largely described differentiation and expansion of granulocytes, which are indeed the responsible for the clinically relevant syndrome, other hematopoietic lineages can be affected. Since both *IDH1* and *IDH2* mutations can occur in early hematopoietic progenitors, DS can involve potentially any lineage (57). Even if there is an overlap between DS and infection related symptoms, if DS diagnostic criteria are present, delaying steroid therapy should be avoided, given that could be life-threatening or fatal. Treatment with empirical antibiotics would be equally important to consider in patients with suspected DS (45).

## Enasidenib and Ivosidenib as Part of Multi-Agents Regimens

Evaluation of the IDH inhibitors in rational combinations are ongoing, with a goal to more fully eradicate the leukemic clones. In this perspective, the clinical benefit of adding ivosidenib or enasidenib to induction, consolidation and maintenance therapy for patients with newly diagnosed *IDH1*/*IDH2*-mutated AML is being evaluated in a phase 3, double-blind, randomized, placebo-controlled study presently recruiting (ClinicalTrials.gov NCT03839771).

In the setting of either newly diagnosed or relapsed/refractory AML patients, elderly or not candidate to intensive chemotherapy, mIDH1/mIDH2 inhibitors could be used in combination with either the hypomethylating agent 5-azacitidine or the bcl-2 inhibitor venetoclax. Indeed, excess D2HG produced by IDH mutant AML blasts confers susceptibility to venetoclax due to ETC dysregulation, suggesting why patients carrying IDH1/IDH2 mutations have more robust responses to the combination treatment of venetoclax and azacitidine. While IDH inhibitors appear to reduce this sensitivity *in vitro*, PDX models and initial clinical trial data of the combination of venetoclax and ivosidenib are encouraging (58, 59).

Results on the phase I multicenter study (NCT02632708), evaluating the safety of enasidenib (100 mg once daily) or ivosidenib (500mg daily) in combination with standard induction (daunorubicin 60 mg/m2/day or idarubicin 12 mg/m2/day for 3 days with cytarabine 200 mg/m2/day for 7 days) and consolidation therapy in 154 newly diagnosed patients with mIDH1 or mIDH2 AML, have been recently published. The frequency of IDH-related DS was low, as expected given the concurrent administration of cytotoxic chemotherapy. There were no significant differences QT interval prolongation among patients treated with ivosidenib or enasidenib, while enasidenib was most frequently associated to increased total bilirubin, consistent with this inhibitor’s known potential to inhibit UGT1A1, without significant clinical consequences.

At the end of induction, In patients receiving ivosidenib (n = 60) or enasidenib (n = CR was achieved respectively in 55% patients treated with ivosidenib and 47% of patients treated with enasidenib; CR/CR with incomplete neutrophil or platelet recovery (CR/CRi/CRp) rates were 72% and 63%, respectively. In patients with a best overall response of CR/CRi/CRp, 16/41 (39%) receiving ivosidenib had IDH1 mutation clearance and 15/64 (23%) receiving enasidenib had IDH2 mutation clearance by digital polymerase chain reaction (50).

Since the synergism in inhibiting differentiation and apoptosis of blast shown in preclinical evaluation of azacitidine combined to ivosidenib, the combination was investigated in an open-label, multicenter, phase Ib trial comprising dose-finding and expansion stages to evaluate the safety and efficacy of combining oral ivosidenib 500 mg once daily continuously with subcutaneous azacitidine 75 mg/m2 on days 1–7 in 28-day cycles in patients with newly diagnosed mIDH1 AML ineligible for intensive induction chemotherapy (ClinicalTrials.gov identifier: NCT02677922). Twenty-three patients received ivosidenib plus azacitidine (median age, 76 years; range, 61–88 years). Treatment-related grade ≥ 3 adverse events occurring in > 10% of patients were neutropenia (22%), anemia (13%), thrombocytopenia (13%), and electrocardiogram QT prolongation (13%). Adverse events of special interest included all-grade IDH differentiation syndrome (17%), all-grade electrocardiogram QT prolongation (26%), and grade ≥ 3 leukocytosis (9%). Median treatment duration was 15.1 months (range, 0.3–32.2 months). The ORR was 78.3%, including 60.9% CR. With a median follow-up of 16 months, median duration of response in responders had not been reached. Median OS has not been reached with a 12-month survival estimate of 82.0% (95% CI, 58.8% to 92.8%). mIDH1 clearance in bone marrow mononuclear cells by BEAMing (beads, emulsion, amplification, magnetics) digital polymerase chain reaction with a sensitivity of 10^-4^ (60) was seen in 10/14 patients (71.4%) achieving CR (43).

These findings led to the development of this combination in a phase 3 double-blind placebo-controlled study of azacitidine with or without ivosidenib (AGILE, ClinicalTrials.gov NCT03173248)^61^ that is currently recruiting in a total of 166 study centers in North America, South America, Asia, and Europe which will provide additional data on efficacy and safety.

## Influence of IDH on FLT3-ITD Status in AML

IDH1 and IDH2 mutations are noted to co-occur with FLT3-ITD mutations in 15%–27% and 8%–30% of AML, respectively (40, 61). Preliminary reports indicate that *IDH1/2m* and FLT3-ITD mutations respond less well to enasidenib or ivosidenib as monotherapy (42, 49).

However, there is no impact of IDH mutation status in newly diagnosed FLT3-ITD+ mutated AML patients. In the first large retrospective study, there was a possible trend of inferior response and survival in dual mutants treated with lower intensity FLT3 inhibitor based regimens, particularly in the older AML population (61). To better understand this unmet clinical need, a retrospective cohort of 91 FLT3-ITD and IDH1 or IDH2 “double-mutated” AML patients at s MD Anderson Cancer Center was evaluated (62). FLT3 and/or IDH inhibitors (FLT3Is and/or IDHIs) were given as a single agent or in combination with cytotoxic chemotherapy or low-intensity therapy (62).

While single-agent FLT3Is and IDHIs demonstrated limited activity, the combination of cytotoxic chemotherapy and FLT3Is resulted in 100% CR+Cri in frontline and 64% in R/R AML patients. Combining FLT3Is with cytotoxic chemotherapy or low-intensity therapy may be effective induction therapy in patients with moderate to high FLT3-ITD allele frequency and/or those with lower IDH allele frequency in the presence of a concomitant FLT3-ITD, because these are likely instances of FLT3-driven disease. However, larger, prospective studies are required to design the optimal personalized approach for FLT3-ITD and IDH1 or IDH2 “double-mutated” AML patients.

## Conclusions

Ivosidenib and enasidenib are promising differentiating agents in the AML therapeutic landscape, that are overall well tolerated with reasonable efficacy as monotherapy agents.

Ongoing and future clinical trials are required to identify the best patient setting and optimize the sequential approach of effective agents to further improve clinical outcome using tailored treatment in AML.

## Author Contributions

All authors: writing—original draft preparation. CC, ND, CD, and HK: supervision. All authors contributed to the article and approved the submitted version.

## Conflict of Interest

The authors declare that the research was conducted in the absence of any commercial or financial relationships that could be construed as a potential conflict of interest.

## Abbreviations

CR, complete remission; cCR, continuous complete remission; CRh, complete remission with partial hematologic recovery; ORR, overall response rate; IDH1, isocitrate dehydrogenases isoform 1; IDH2, isocitrate dehydrogenases isoform 2; NPM1, nucleophosmin; FLT3, fms like tyrosine kinase 3; ITD, Internal Tandem Duplication; 2HG, 2-hydroxyglutarate; D2HG, D-2-hydroxyglutarate; αKG, α-ketoglutarate; HOXA, homeobox; NADPH, nicotinamide adenine dinucleotide phosphate; ALKBH, α-KG-dependent alkB homolog; TET, ten-eleven translocation methylcytosine dioxygenases; TET2, Tet methylcytosine dioxygenase 2; HSPC, hematopoietic stem and progenitor cells; DS, differentiation syndrome; R/R AML, relapsed/refractory acute myeloid leukemia; RTK, receptor tyrosine kinase; TD, transfusion dependence; TI, transfusion independence; VAF, variant allelic frequence.
